# Characterization of the erythropoietin/erythropoietin receptor axis in a rat model of liver damage and cholangiocarcinoma development

**DOI:** 10.1007/s00418-012-1037-x

**Published:** 2012-10-04

**Authors:** Federico Moriconi, Pierluigi Ramadori, Frank C. Schultze, Martina Blaschke, Ahmad Amanzada, Sajjad Khan, Giuliano Ramadori

**Affiliations:** 1 Department of Gastroenterology and Endocrinology, Center of Internal Medicine, University of Göttingen, Robert Koch Straße 40, 37075 Göttingen, Germany; 2 Department of Cell Physiology and Metabolism, University Medical Centre of Geneva, Geneva, Switzerland

**Keywords:** Cholangiocarcinoma (CC), Chronic liver injury, Erythropoietin (EPO), Erythropoietin receptor (EpoR), Thioacetamide (TAA)

## Abstract

It has been recently shown that the biological effects of erythropoietin (EPO) are not limited to the hematopoietic compartment but, as pleiotropic glycoprotein, this hormone can exert pro-angiogenic and tissue-protective functions also in a wide range of non-hematopoietic organs. The role of EPO and the effective functionality of its receptor in solid tumors are still a controversial point of debate. In the present work we analyzed the gene expression of EPO and its cognate receptor (EpoR) in a rat model of thioacetamide-induced damage and tumor. An analysis of the EPO/EpoR axis was also performed on human cholangiocarcinoma (CC) cell lines. A progressive increase of EPO and EpoR mRNA can already be observed during the fibrotic–cirrhotic development with a peak of expression rising at tumor formation (24.7 ± 9.9-fold increase and 15.5 ± 1.1-fold increase, respectively, for the two genes). Co-localization studies by immunofluorescence revealed hepatocytes in the regenerative cirrhotic nodules (Hep Par-1^+^) and in the dysplastic bile duct cells (CK19^+^) as the major EPO producers in this specific condition. The same cell populations, together with endothelial cells, exhibited an increased expression of EpoR, although all the non-parenchymal cell populations in the liver exhibited modest basal mRNA levels. Challenging human CC cells, Mz-Cha-2, with a combination of EPO and SCF resulted in a synergistic effect on the gene expression of EPO, CyclinD1 and PCNA. This study suggests that the autocrine and paracrine release of endogenous EPO in the microenvironment may contribute to the development and maintenance of the CC possibly in cooperation with other signaling pathways.

**Electronic supplementary material:**

The online version of this article (doi:10.1007/s00418-012-1037-x) contains supplementary material, which is available to authorized users.

## Introduction

The biological effects of erythropoietin (EPO) are not only limited to the hematopoietic system, but also an increasing body of evidences has recently focused attention on a wide spectrum of functions in several non-hematopoietic organs (reviewed in Lombardero et al. 2011). Indeed, the presence of EPO and its cognate receptor (EpoR) has been documented in many non-hematopoietic cell types, such as vascular endothelial cells, smooth muscle cells, cardiac myocytes, neurons and macrophages (Brines and Cerami 2012). In addition to stimulating erythropoiesis, EPO has been reported to promote angiogenesis, vascular stability and endothelial cell survival (Ribatti et al. 2003). EPO has also been suspected of procancerogenic activity.

It has been postulated that, if tumor cells express EpoR, erythropoiesis-stimulating agents could activate these receptors to induce tumor cell proliferation (Hadland and Longmore 2009). Moreover, several cancer cells, such as breast carcinoma, endometrial carcinoma and lung carcinoma cells (Hedley et al. 2011), have been described as being able to synthesize EPO and to express the EpoR on their surface suggesting a possible autocrine or paracrine effect of the hormone on cell survival and proliferation. However, the functional role of EPO in tumor biology is still far from being fully understood. In spite of a growing amount of in vitro and in vivo studies (Shi et al. 2010; Kumar et al. 2011) that associate the presence of EpoR with the promotion of cancer cells proliferation and invasion, a linear correlation between EpoR activation and an efficient responsiveness to exogenous EPO administration has not been established.

At the bedside, the therapeutic benefit of the clinical use of erythropoiesis-stimulating agents in cancer is still a delicate controversial discussion point. Indeed, cancer-related and chemotherapy-induced anemia that represents a further negative variable in the quality of life for many cancer patients prompted clinicians to use EPO and EPO derivatives in several clinical trials (McKinney and Arcasoy 2011). Although in certain conditions it was effective in correcting the hematocrit and hemoglobin levels, detrimental effects on tumor progression and increased mortality have been reported in a considerable number of clinical studies (Szenajch et al. 2010).

Previous clinical reports have recorded erythrocytosis and an increase in EPO serum levels in patients with hepatocellular carcinoma (Cheng et al. 2002), whereas histological evidences of augmented EPO synthesis have been identified from the analysis of hepatic cancer samples and liver tissue surrounding the tumor (Ribatti et al. 2007). So far, the EPO/EpoR system has been indicated as playing a major angiogenetic role in murine models of hepatocarcinogenesis (Nakamatsu et al. 2004) and in vitro studies have indicated the presence of mRNA and protein of both genes in HepG2 and Hep3B hepatoma cell lines. However, no effect on cell proliferation or invasion has been observed after exogenous EPO administration (Farrell and Lee 2004).

Cholangiocarcinomas (CC) refer to malignancies of the biliary tree epithelia. They are relatively rare cancers (about 3 % of malignant tumors) in western countries (USA and Europe), but remain difficult to diagnose and treat, respond very poorly to chemotherapy and radio-therapy and have a high mortality rate: over 50 % in untreated patients within a few months of diagnosis (Patel 2011).

The present study offers a further insight into the tumor biology of the EPO/EpoR axis, with a particular focus on chronic liver injury and its progression to CC. With the current experimental model, we not only analyzed the progressive changes in EPO gene expression and protein production during chronic liver damage and tumor development, but also attempted to identify the cellular sources and the probable targets of the hematopoietic hormone. Finally, we pursued the characterization of functional effects of EPO on proliferation and growth of human CC cell lines, also in combination with the hematopoietic growth factor SCF that has been previously identified in the same cell populations together with its receptor c-kit. A possible synergistic effect on genes involved in cell cycle regulation interestingly emerged by challenging CC cell lines with a combination of growth factors.

The aberrant expression of EPO and EpoR described in this rat model of CC and confirmed in human cell lines suggests a possible local autocrine and paracrine release of the hormone not only for direct tumor survival and proliferation, but also for the hepatocytes’ response to chronic liver damage, possibly in a synergistic regulatory mechanism with other growth factors.

## Materials and methods

### Animals and experimental model

Male Sprague–Dawley (SD) rats weighing 330–370 g were used in these experiments. The animals were obtained from Charles River (Sulzfeld, Germany) and Harlan Winkelmann (Borchen, Germany) and received human care according to the guidelines of the local institution and the National Institutes of Health. We adhered to the institutional policies and the relevant guidelines for care and use of laboratory animals. All animal experiments were approved by the ethics review board, and were continuously supervised by the local ethic commission. The animals were divided into five groups, including a control group for each time point and experimental groups following a time progression up to 18 weeks. Rats were housed in an animal room with a 12:12-h light–dark cycle, with food and water available ad libitum. CC was induced according to the protocol of Yeh et al. (2004) with a slight modification. The experiment group received 500 mg/l thioacetamide (TAA) (Sigma Aldrich) in their drinking water every day up to the time they were killed. Four control and five TAA-treated rats were killed every fourth week during the study to examine the effect of TAA. As 80 % of TAA-treated rats developed CC at week 16, the experiment was stopped at week 18, at which time 100 % of the TAA-treated rats had developed CC.

### Immunohistochemical analysis

Five micrometer cryostat sections were evaluated by immunohistochemistry (Reichert Jung, Wetzlar, Germany) with rabbit anti-EPO and rabbit anti-EpoR (Santa Cruz Biotech), mouse anti-Hep Par-1 (Dako), mouse anti-CK19 (Novocastra), mouse anti-ED-2 (Serotec) and mouse anti-alpha-SMA antibodies (Sigma). Cryostat sections were air dried and used for immunohistochemical studies after fixation for 10 min in acetone at room temperature. After blocking of non-specific binding with 1 % bovine serum albumin (Serva, Heidelberg, Germany) and 10 % goat serum (DAKO, Glostrup, Denmark) containing PBS (Biochrom, Berlin, Germany) for 1 h at room temperature, the sections were incubated overnight at 4 °C with a single or two primary antibodies simultaneously. Antigens were visualized using two different immunohistochemical methods. For light microscopy, the standard alkaline phosphatase anti-alkaline phosphatase (APAAP)/Fast Red method was used as described previously (Neubauer et al. 2008). Double immunostaining for studies of antigen co-localization was performed using fluorescent conjugates. The rabbit polyclonal and mouse monoclonal antibodies were detected with Alexa-555-conjugated goat-anti-rabbit and Alexa-488 conjugated goat-anti-mouse secondary antibodies (Molecular Probes, Leiden, Netherlands). Sections were counter-stained with DAPI (Molecular Probes) and observed with an epifluorescence microscope (Axiovert 200 M, Zeiss, Jena, Germany). Negative control immunostainings were performed by omitting the primary antibody and using isotype-matching control immunoglobulins.

### Total protein isolation and Western blot analysis

Liver tissue samples frozen in liquid nitrogen at several time points were lysed in lysis buffer (0.50 M Tris HCl pH 7.6, 150 mM NaCl, 10 mM EDTA, 1 % Triton X-100, 1 mM PMSF and 10 μl/ml cocktail proteases inhibitors) and incubated for 30 min on ice. After 15 min of centrifugation at 12,000 rpm, the protein concentration in the supernatant was determined with the Coomassie method (Pierce, Rockford, IL). 50 μg of total protein-containing samples was loaded on a 4–12 % NUpage Bis–Tris gel (Invitrogen) and separated by electrophoresis followed by protein transfer onto a nitrocellulose membrane according to the manufacturer’s instructions. Immunodetection was performed according to the ECL-Western blotting protocol. The primary antibodies mouse anti-EPO (Acris Antibodies), rabbit anti-EpoR (Santa Cruz Biotech; M20) and mouse anti-EpoR (clone 3D10, Sigma) were used in a 1:200 dilution. Beta-actin, and peroxidase-labeled anti-mouse and anti-rabbit secondary antibodies were each used in a 1:1,000 dilution.

### Isolation of different liver cell populations from adult control rats and culture conditions

Hepatocytes were isolated from healthy adult rats by means of collagenase treatment in a recirculating in situ perfusion technique and cultured as reported previously (Ramadori et al. 2010a). Hepatic stellate cells (HSC) and myofibroblasts were isolated by sequential in situ perfusion with collagenase and pronase (Piscaglia et al. 2009). After perfusion with collagenase, hepatocytes were removed by low-speed centrifugation. Non-parenchymal cells were separated from cellular debris and erythrocytes by density centrifugation in a Nycodenz gradient. Sinusoidal endothelial cells (SECs) and Kupffer cells were obtained as reported earlier (Armbrust et al. 2004).

### Culture and stimulation of human CC cell lines

Mz-Cha-2 and EGI-1 were cultured in a DMEM medium, whereas TFK-1 CC cells were cultured in an RPMI medium as reported previously. Cells were starved overnight by serum depletion before stimulation analyses. SCF and EPO recombinant human proteins were used at a concentration of 10 ng/ml and 10 U/ml, respectively, alone or in combination for 24 h. After stimulation with growth factors, the cells were harvested for protein and RNA preparation. In the control groupm, cells were harvested at the same time point without adding growth factors. At 24 and 48 h, a dose–response curve was obtained for EPO treatment at concentrations of 0.1, 1 and 10 U/ml.

### RNA isolation and real-time PCR analysis

RNA was isolated from the rat livers and the isolated cell populations as described previously (Ramadori et al. 2010b). Reverse transcription of RNA into cDNA was performed with the Superscript^®^ kit (Invitrogen), according to the manufacturer’s instructions. Real-time PCR analysis of cDNA was performed with an Applied Biosystems Step One Plus^®^ Sequence detection system using Fast Taq Sybr Green^®^ (AB, Applied Biosystems) and the primers used are listed in Table 1. The primers were synthesised by Invitrogen. The quantity of the PCR products of the genes of interest (*Q*) was normalized to the housekeeping gene. Fold change expression was calculated using the threshold PCR cycle values of the housekeeping gene Ubiquitin C (UBC*)* (Ct_UBC_), using the formula: *Q* = 2^−∆Ct^, where ∆Ct = Ct_target_−Ct_UBC_, and β-actin for rat samples, whereas β-actin and GADPH for human samples. The quantities of normalized PCR products detected in the livers of treated rats were compared to the values in control rats, and the relative expression was plotted against the observation time. In all cases, two or three series were analyzed in duplicate.

**Table 1:** Real-time RT-PCR primers used in the study

Name	Forward	Reverse
rno EPO	CCA GCC ACC AGA GAG TCT TC	TGC AGA AAG TAT CCG CTG TG
rno EpoR	ACA CGT CGA GTT TTG TGC CA	TGG ATG ATG CGG TGG TAG C
rno β-actin	TGT CAC CAA CTG GGA CGA TA	AAC ACA GCC TGG ATG GCT AC
rno UBC	CAC CAA GAA CGT CAA ACA GGA A	AAG ACA CCT CCC CAT CAA ACC
hs EPO	CTC CCT CAC CAA CAT TGC TT	GGC CCT GTG ACA TCC TTA GA
hs EPOR	TAC AGA GGG TGG AGA TCC	GAT CTT CTG CTT CAG AGC C
hs EpoR-s	GGA GCC AGG GCG AAT CAC AGG	GCC TTC AAA CTC GCT CTC TG
hs CyclinD1	AAC TAC CTG GAC CGC TTC CT	CCA CTT GAG CTT GTT CAC CA
hs PCNA	GCT GTG TAG TAA AGA TGC CT	TAT GGT AAC AGC TTC CTC CT
hs Ki67	CCA GAG GAA GTA TTC CTA CAG	CCC TCA CTC TCA TTA ATG GA
hs GADPH	AGC CCA GAA CAT CAT CCC TG	CCC CAC CTT CTT GAT GTC ATC
hs β-actin	CTG GCA CCC AGC ACA ATG	CCG ATC CAC ACG GAG TAC TTG

### Statistics

Descriptive statistics, *t* test, and two-way ANOVA for dependent and independent samples and graphs were performed with Statistica 6.0 (StatSoft, Hamburg, Germany). We used a constant level of *P* = 0.05 for rejection of the null hypothesis.

## Results

### Aberrant gene expression of the EPO/EpoR axis during chronic liver damage and CC development

**Fig. 1:**
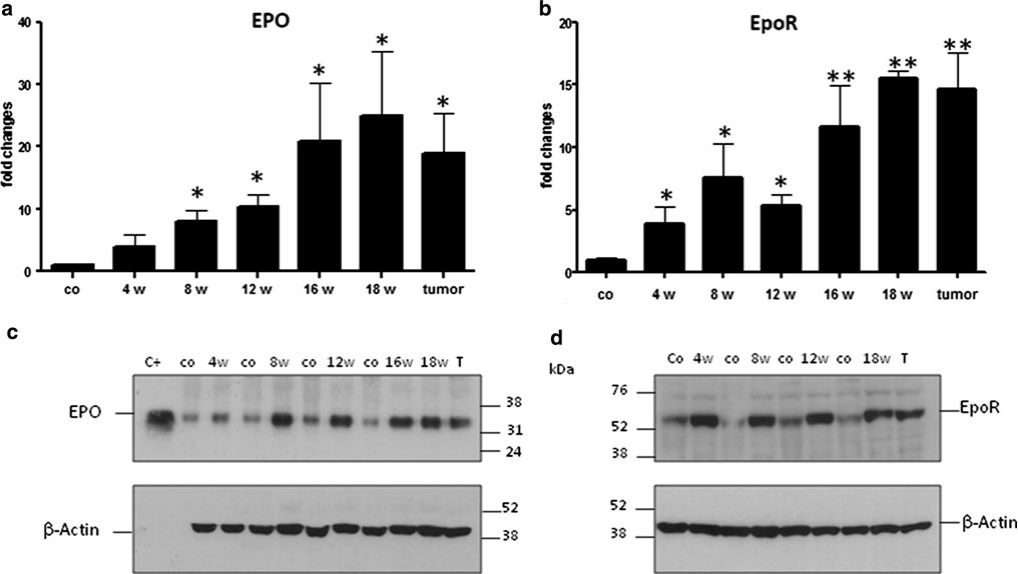
mRNA and protein expression levels of EPO and EpoR in TAA-treated rats. **a** Number of EPO RNA transcripts expressed in fold changes relative to Ubiquitin C gene expression (*t* test statistical analysis was performed at each time point vs. control group, *P < 0.05 and **P < 0.01, *N* = 4/5). **b** Number of EpoR RNA transcripts expressed in fold changes relative to Ubiquitin C gene expression (*t* test statistical analysis was performed at each time point vs. control group, *P < 0.05 and **P < 0.01, *N* = 4/5). Representative Western blot for EPO (**c**) and EpoR (**d**) protein levels in total liver and excised tumors protein extracts. *Co* indicates respective rat control liver for every time point analyzed. Recombinant rat EPO was used as positive control (C+)

As indicated in the RT-PCR analyses, the expression of EPO mRNA progressively increased up to 24.7 ± 9.9-fold reaching a maximum at week 18 and 16.1 ± 3.5 in the isolated CC (extracted tumor tissue) from 18 weeks TAA-treated livers (*P ≤ 0.05) (Fig. 1a). Interestingly, already after 4 weeks of treatment, the expression started to increase and became statistically significant at week 8, therefore indicating an EPO overproduction preceding the appearance of tumor. This early increase was clearly reproduced also at the protein level in the Western blot images (Fig. 1c). In fact, the EPO band (~34 kDa) showed an increase already at 4 weeks after the treatment became progressively stronger after 8 weeks of treatment, and persisted until the cancer nodules appeared. Recombinant rat EPO was used as positive control (Fig. 1c).

The mRNA levels of EpoR showed a similar and parallel up-regulation with a maximum at week 18 (15.5 ± 1.1-fold) (*P ≤ 0.05). Furthermore, EpoR mRNA expression analyzed in the excised tumor was 14.6 ± 2.9-fold higher than in the normal liver (Fig. 1b). The immunoblot for EpoR (~56 kDa) showed a clear increase in the signal already after 4 weeks of treatment and remained strongly expressed at all time points analyzed (Fig. 1d).

### Identification of EPO-producing cells in rat liver during TAA administration

**Fig. 2:**
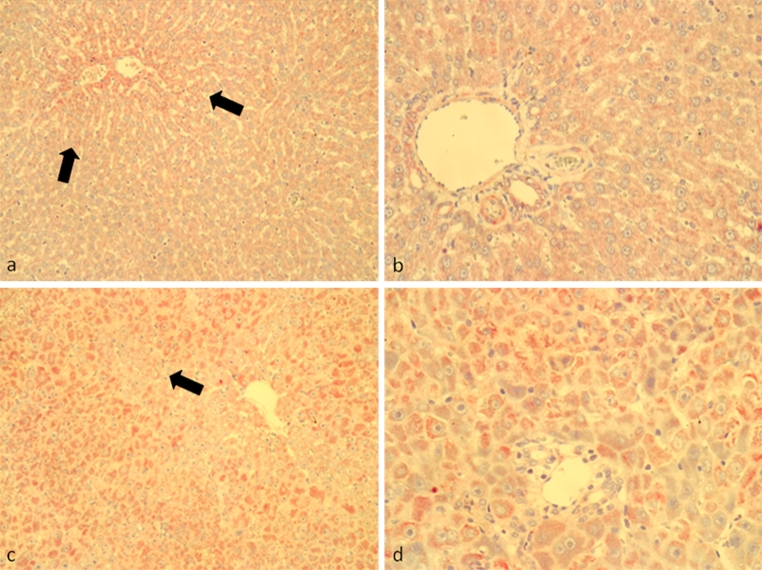
Immunohistochemical detection of EPO with alkaline phosphatase anti-alkaline phosphatase (APAAP)/Fast Red method in rat liver sections. **a** Perivenular (×100) and **b** periportal (×200) spaces in normal control livers. EPO was moderately expressed in hepatocytes surrounding perivenular spaces (*large arrows*). **c** Perivenular and **d** periportal spaces in 4 weeks TAA-treated rats (×100 and ×200, respectively)

**Fig. 3:**
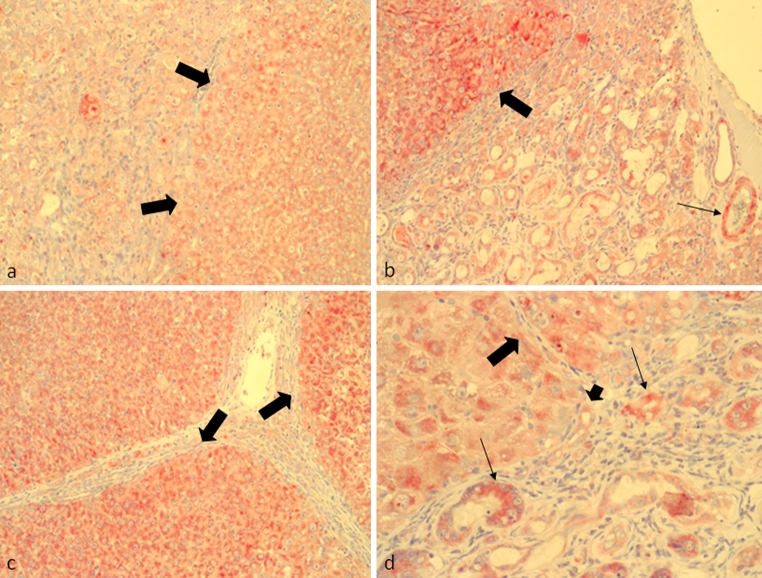
Immunohistochemical detection of EPO with alkaline phosphatase anti-alkaline phosphatase (APAAP)/Fast Red method in rat liver sections. **a**, **b** Internodular (×100 and ×200) spaces of 8 weeks TAA-treated rats and **c**, **d** of 12 weeks TAA-treated rats. At this time point, EPO positivity is mainly localized in the regenerating nodules inter-septa (*large arrows*) and in isolated hyperplastic bile ducts (*thin arrows*). The presence of EPO-negative bile ducts is indicated by *short arrow*

**Fig. 4:**
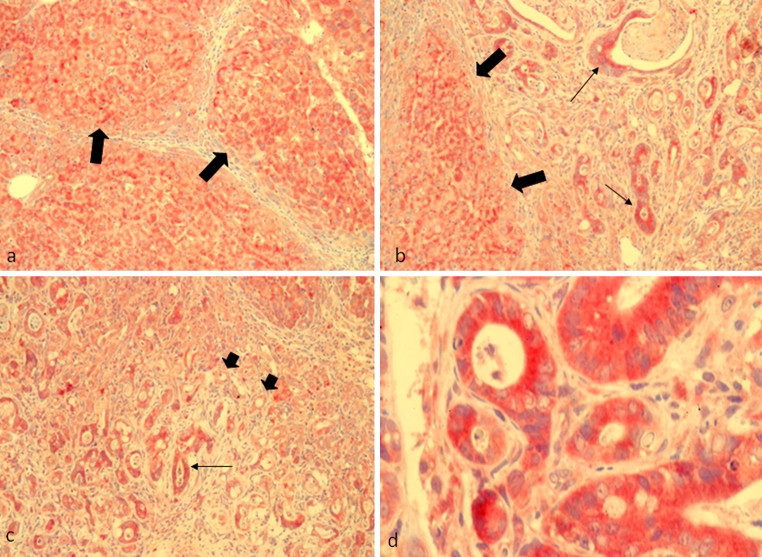
Immunohistochemical detection of EPO with alkaline phosphatase anti-alkaline phosphatase (APAAP)/Fast Red method in rat liver sections. **a**, **b** Three regenerative nodules (×100 and ×200) separated by (negative) fibrotic septa of 16 weeks TAA-treated rats and **c**, **d** a particular part of the tumor area of 16 weeks TAA-treated rats. EPO positivity is mainly localized in the regenerating nodules intersepta (*large arrows*) and hyperplastic bile ducts (*thin arrows*). Not all proliferating bile ducts seem to participate in EPO production (**c** *short arrows*). **d** Details of a CC area at higher magnification (×400)

According to previous studies (Weidemann and Johnson 2009), EPO production in the normal liver might be attributed mainly to the hepatocytes surrounding the central veins (Fig. 2a, b), although recent unpublished data from our group suggest Kupffer cells and HSC as possible contributors to hepatic EPO production. The immunostaining reported here showed a progressive increment in EPO synthesis that already rose up at early stages after TAA treatment (4–8 weeks) (Figs. 2c, d, 3a, b). As the fibrotic process progressed (Fig. 3c, d), the isolated nodules of regenerating hepatocytes between the fibrotic septa revealed an increased positivity that is well illustrated in the APAAP images obtained at weeks 8 (Fig. 3a, b), 12 (Fig. 3c, d) and 16 (Fig. 4a, b). Gradually, EPO-positive bile ducts secondarily appeared, although not all the proliferating bile ducts seemed to participate in EPO production, as clearly shown in the histological analyses of Fig. 3d (short arrows). In fact, the EPO expression in cholangiocytes grew in a time-dependent manner and became particularly elevated in the hyperplastic bile ducts of the cancer tissue, where it was, however, possible to individuate isolated clusters of negative bile ducts (Fig. 4c, d).

### Immunolocalization of the EpoR in the rat liver during TAA administration

**Fig. 5:**
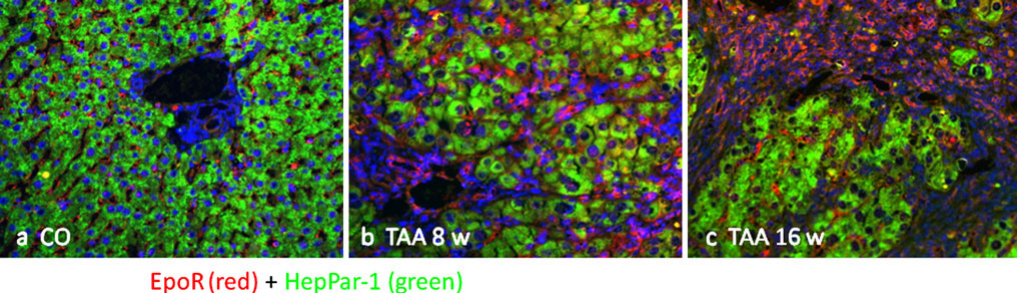
Co-localization image of a double immunofluorescence stain for EpoR (*red channel*) and the hepatocyte-specific marker Hep Par-1 (*green channel*). Representative images of periportal spaces of **a** normal control, **b** 8-week-treated and **c** 16-week-treated TAA rat livers at ×200. Sections were analyzed with an epifluorescence microscope and the images were obtained with AxioVision system software

**Fig. 6:**
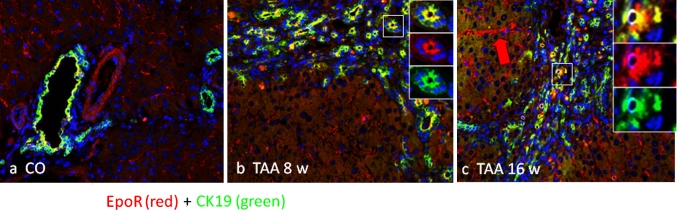
Co-localization image of a double immunofluorescence stain for EpoR (*red channel*) and the cholangiocyte-specific marker CK19 (*green channel*). Representative images of periportal spaces of **a** normal control, **b** 8-week-treated and **c** 16-week-treated TAA rat livers at ×200. In **b** and **c** it is possible to appreciate the detailed merge images of double positive (CK19/EpoR) bile ducts and a clear intra-nodular sinusoidal positivity (**c** *red arrow*). Sections were analyzed with an epifluorescence microscope and images were obtained with AxioVision system software

**Fig. 7:**
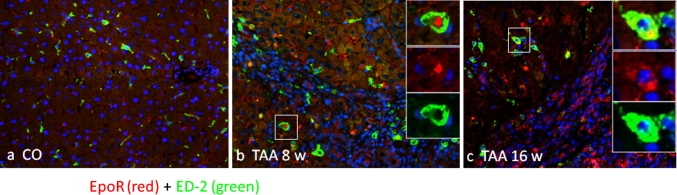
Co-localization image of a double immunofluorescence stain for EpoR (*red channel*) and the macrophage-specific marker ED-2 (*green channel*). Representative images of periportal spaces of **a** normal control, **b** 8-week-treated and **c** 16-week-treated TAA rat livers at ×200. At higher magnification, merged images indicate a possible co-localization of EpoR with ED-2. Sections were analyzed with an epifluorescence microscope and images were obtained with AxioVision system software

**Fig. 8:**
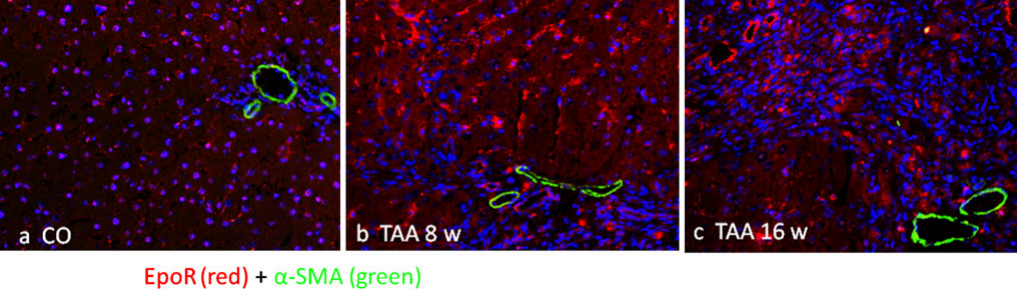
Co-localization image of a double immunofluorescence stain for EpoR (*red channel*) and alpha smooth muscle actin (*green channel*). Representative images of periportal spaces of **a** normal control, **b** 8-week-treated and **c** 16-week-treated TAA rat livers at ×200. No evident co-localization signals were detected. Sections were analyzed with an epifluorescence microscope and images were obtained with AxioVision system software

As presented in Fig. 5, no evident co-localization of EpoR and Hep Par-1 was detectable by immunostaining, indicating a very low expression of the receptor in rat hepatocytes (Fig. 5a, c). In normal liver, rare bile ducts and hepatic vessels showed a weak positivity for EpoR antibody (Figs. 5a, 6a). During chronic injury, increased EpoR protein expression was localized in the sinusoids of the regenerating nodules (Fig. 5b, c), suggesting an important role for tissue vascularization, and in the CK19^+^ hyperplastic cells of the CC (Fig. 6b, c). Moreover, merge signals co-localizing with EpoR antibodies have also been identified in ED-2 positive cells, although the sinusoidal location hardly allows discrimination with endothelial cells (Fig. 7). No alpha-SMA positive cells were found to be positive for EpoR antibody (Fig. 8).

### EPO and EpoR mRNA expression in isolated rat liver cells

**Fig. 9:**
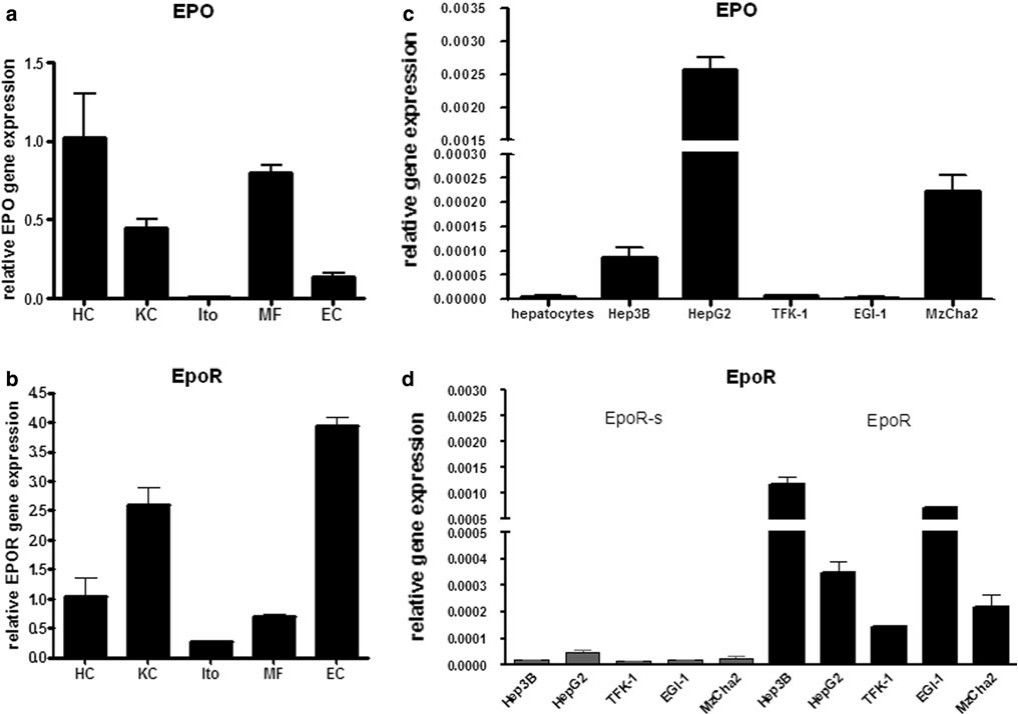
EPO (**a**) and EpoR (**b**) gene expression in liver parenchymal and non-parenchymal cells isolated from normal rat livers compared to rat primary hepatocytes. Relative levels of EPO (**c**) and EpoR (**d**) gene expression in different human hepatoma and CC cell lines

To confirm previously described in vivo observations, we analyzed the expression of EPO and EpoR in freshly isolated parenchymal and non-parenchymal rat liver cells. As expected, under normoxic and un-stimulated conditions, the hepatocytes expressed only modest levels of both EPO and EpoR gene expression as absolute value. Although RNA levels were detectable in all studied cell populations, hepatocytes and myofibroblasts showed the highest EPO gene expression levels (Fig. 9a), while EpoR gene expression was predominantly seen in isolated Kupffer cells and endothelial cells (Fig. 9b).

### EPO and EpoR mRNA expression in different human cell lines

Considering in advance that CK-19^+^ cells were EpoR^+^ by performing immunohistochemistry analysis (Fig. 6), we decided to employ human CC and HCC cell lines for our in vitro studies. The number of EPO gene transcripts in hepatoma and specifically in the human CC cell line Mz-Cha-2 increased significantly compared to normal hepatocytes (Fig. 9c). Whereas other CC cell lines presented expression levels similar to primary hepatocytes, quantification by PCR revealed a particularly high expression in Mz-Cha-2, although the HCC cell line HepG2 showed the highest number of transcripts in un-stimulated conditions (Fig. 9c).

Analysis of the EpoR gene was conducted for the two transcripts that code for different peptide isoforms of the receptor, the full-length (EpoR) and the soluble form (EpoR-s) derived from alternative splicing (Arcasoy et al. 2003). The identification of the two clones enabled a selective PCR analysis in human cell lines. Interestingly, EpoR gene expression was plainly detectable in these cell lines, whereas EpoR-s expression was barely detectable (Fig. 9d).

### Changes of EpoR gene expression after SCF treatment in combination with EPO in different human CC cell lines

**Fig. 10:**
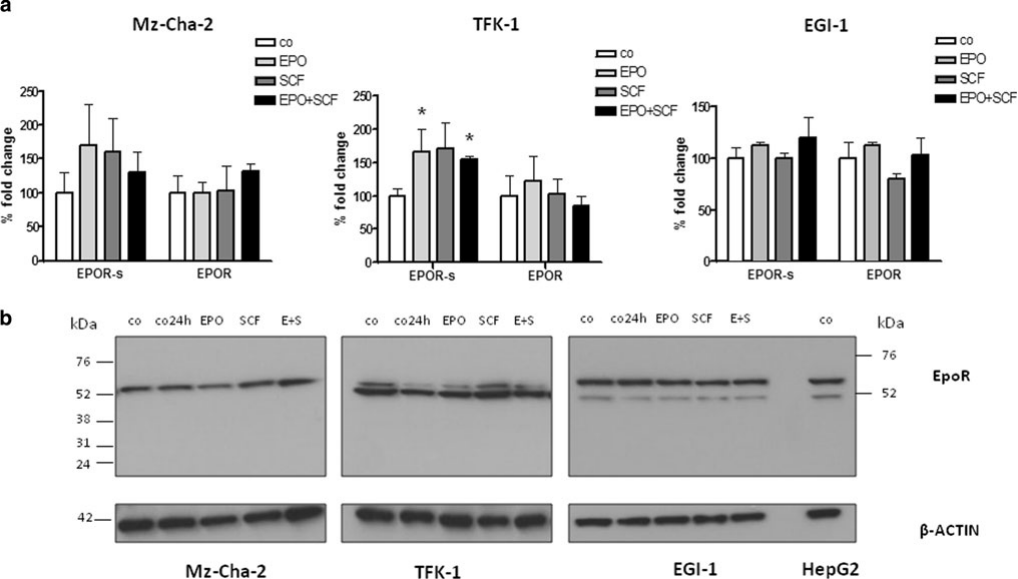
EpoR gene expression in three CC cell lines challenged with EPO (10 U/ml), SCF (10 ng/ml) or a combination of both growth factors for 24 h under serum-free conditions. **a** Gene expression of different EpoR isoforms [full length (EpoR) and soluble (EpoRs)] was analyzed in all three indicated CC cell populations following growth factor challenge (Mz-Cha-2, TFK-1, and EGI-1). **b** Western blot analysis performed after cell stimulation indicates the presence of EpoR protein in all cell lines using an EpoR antibody for the extracellular domain. *Co* indicates control cells in un-stimulated conditions and not starved, whereas *Co 24* *h* refers to untreated starved control cells. HepG2 protein extract was used as a positive control

We investigated also the effects of SCF (c-kit ligand), a growth factor that we described in the same experimental model (Mansuroglu et al. 2009) and that was previously identified in the same cell lines (Kamenz et al. 2006). We analyzed the human CC cell line Mz-Cha-2 and found no significant changes of EpoR or EpoR-s gene expression after stimulation with the growth factors EPO and SCF, alone or in combination (Fig. 10a). We performed the same experiments in the other CC cell lines, TFK-1 and EGI-1 (Fig. 10a). While in this last cell population (c-kit negative cells, Shi et al. 2010) we were unable to detect significant changes for the same genes following growth factor stimulation (data not shown) in spite of an evident presence of EpoR protein (Fig. 10b), surprisingly in TFK-1 we observed a decrease in EPO production in parallel with a decrease in CyclinD1 and PCNA expression after treatment with a combination of SCF and EPO (data not shown), in contrast to a significant increase of EpoR-s transcripts (Fig. 10a). Beyond increasing the level of complexity, this result suggests a diverse responsiveness of CC cells based on the presence of different growth factors in the extracellular milieu.

To investigate the presence of different EpoR isoforms, i.e., a soluble- (EpoR-s), truncated (EpoR-t) and a full-length (EpoR-f) isoform (here indicated as EpoR), we performed a Western blot analysis using an EpoR-specific antibody (3D10 clone) recognizing the extracellular domain of the receptor (Supplementary Fig. 1). EpoR protein levels were detectable only for EpoR as shown in Fig. 10b (~56 kDa). In every cell population, we could not detect EpoR-s (~28 kDa) or EpoR-t (~35 kDa) isoforms (Fig. 10b).

### SCF treatment in combination with EPO induced significant changes in EPO gene expression and of cell cycle genes on human Mz-Cha-2 cell line

**Fig. 11:**
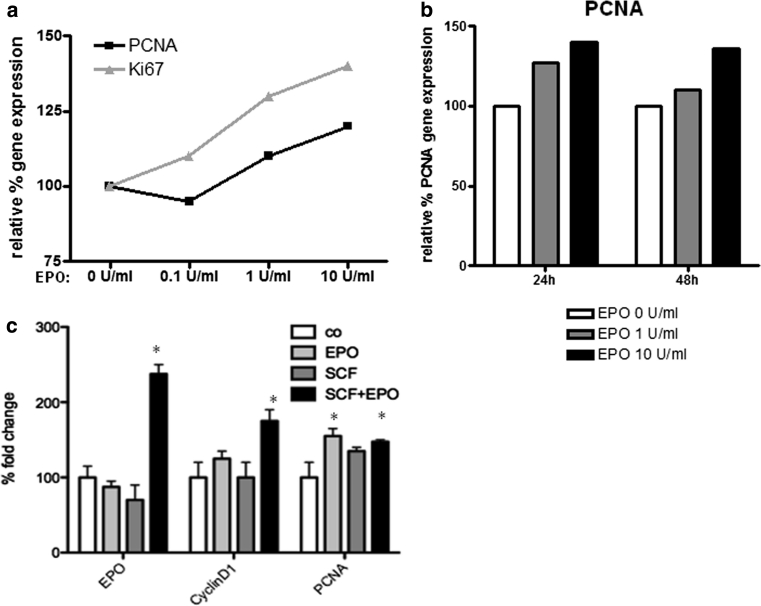
Cell proliferation gene expression in Mz-Cha-2 cells challenged for 24 h with EPO (10 U/ml), SCF (10 ng/ml) or a combination of both. **a** Ki67 and PCNA gene expression was evaluated after a dose-dependent challenge with recombinant human EPO. **b** PCNA expression was analyzed in a range of 48 h using two different EPO concentrations (1 and 10 U/ml). **c** EPO responsiveness of Mz-Cha-2 was investigated by analyzing EPO, CyclinD1 and PCNA gene expression after 24 h of incubation with the growth factors as indicated above

The functional activity of the EPO/EpoR axis was verified in Mz-Cha-2 cell line which was shown to express the highest EPO levels under normoxic condition. Firstly, a dose-dependent assay of the effect on cell cycle gene expression was performed with different concentration of EPO at 0.1, 1 and 10 U/ml and evaluated 24 h after the treatment. The results indicated a dose-dependent response of Ki67 and PCNA genes (Fig. 11a). Over a period of 48 h, EPO at a concentration of 10 U/ml enhanced PCNA gene expression in Mz-Cha-2 compared to untreated controls (Fig. 11b).

We also investigated the effects of SCF in the same cell line. Interestingly, although the stimulation with SCF alone did not show any significant increase in CyclinD1 and PCNA gene expression, challenging Mz-Cha-2 cells with a combination of EPO and SCF resulted in a significant increase in EPO gene expression (more than 100 % increase) and in a significant 70 % augmentation of CyclinD1 and 50 % of PCNA (Fig. 11c).

## Discussion

The existence of the EPO/EpoR signaling pathway has been recently observed in a wide range of tumors and cancer cell line (Sinclair et al. 2007), but its role and true function in the context of cancer biology are still a matter of controversy and currently under investigation. Although classical investigation of partial hepatectomy indicated an important synthesis of the hormone accompanying liver regeneration and restoration of the liver mass (Bockhorn et al. 2008) and recent studies revealed that an increased EPO gene expression was associated with promotion of synaptic plasticity in an experimental model of nervous system injury (Mengozzi et al. 2012), the importance of this hormone in this particular context is still underestimated.

Overproduction of EPO has been described in several tumor conditions among others in the liver, where it has been associated mainly with its angiogenic properties (Ribatti et al. 2007). However, the presence of the EpoR on tumor cell lines has led to disparate interpretations. In fact, whereas many studies have reported an increase in EPO levels in terms of protein expression, the lack of specificity of commercially available antibodies has limited the understanding of a potential functional role of this pathway under these pathological conditions (Fandrey 2008). The hepatic reactivation of EPO production has been shown to occur in several clinical and experimental pathological conditions, such as acute phase response and acute liver injury and repair (Ramadori et al. 2010a), but the mechanisms and the physiological consequences of this switch remain unclear.

In the present work, we investigated the expression of the hormone and its receptor in an experimental model of chronic liver injury that leads to the development of intra-hepatic CC. In detail, the chronic administration of TAA in drinking water has been shown to induce hepatic injury with periportal fibrosis that evolves to cirrhosis and end-stage neoplastic transformation of the hyper-proliferative bile ducts within the stromal tissue of the portal spaces (Mansuroglu et al. 2009).

We analyzed EPO production by evaluating gene expression and confirming it by protein detection. Its synthesis and production increased progressively up to 18 weeks as illustrated by mRNA expression and Western blot analysis. Immunohistochemistry indicated a localization of the hormone that was particularly abundant in hepatocytes of the regenerating cirrhotic nodules and in the dysplastic proliferating bile ducts that were progressively transforming. The metabolic increase required for proliferation and possibly the altered oxygen tension might reasonably explain the high expression levels of EPO seen already at 4 weeks after treatment. In line with these observations, it is also interesting to mention a recent study on EPO expression during tumor development in which tumor-derived PDGF-BB was shown to induce a dramatic increase of circulating EPO responsible for tumor growth promotion, angiogenesis and hematopoiesis. Importantly, the major sources of EPO in these specific conditions were identified in liver and spleen, but not in the kidneys (Xue et al. 2012).

EPO gene expression during hypoxia and the high expression levels observed in hepatoma cell lines have been classically associated with the activation of the transcription factor family HIFs, hypoxia-inducible factor. In this context, evidences concerning the direct transcriptional regulator of the EPO gene in the liver (HIF-1 or HIF-2) are still the subject of intense debate. However and interestingly, a recent genomic report by Steinmann et al. (2011) conducted on several cancer cell lines identified the hepatoma cell line Hep3B as the tumor cell population with the highest EPO expression in a wide range of cells, higher even than renal cancer cells. Of note, Steinmann et al. were able to demonstrate that this peculiarity of gene expression might be due to the lack of methylation of the EPO promoter in this cell population, indicating a further mechanism of transcriptional control. In fact, our analyses revealed a higher expression of EPO transcripts in normal HepG2 cells compared to Hep3B, and we were able to collocate the CC cell line Mz-Cha-2 among the different examined CC cell lines expressing the highest levels of EPO in absolute value.

We used the polyclonal antibody (M-20) previously shown to have a reliable specificity (Lopez et al. 2011) to identify the EpoR by Western blot and immunohistochemistry, since the anti-EpoR antibody (C-20 Santa Cruz Biotech) has been reported to cross-react with HSP70, which is also highly expressed in tumor tissue (Brown et al. 2007). Interestingly, the detection of an approximately 59 kDa band by Western blot using the polyclonal M-20 antibody suggested the significantly overexpressed presence of the EpoR in liver tissue during CC development, as also confirmed by RT-PCR analysis.

A recent study by Khankin et al. (2010) reported the presence of a soluble form of EpoR (EpoR-s) in the serum of dialysis patients detectable as a 28 kDa protein resulting from an alternative mRNA splicing. Interestingly, the expression of EpoR-s was shown to be induced by pro-inflammatory cytokines and its circulating levels were correlated with increased peripheral EPO resistance. In contrast, although we could detect a very low number of EpoR-s transcripts in the CC cells analyzed in our study, we could not identify any bands other than a 56 kDa by Western blot using a reliable monoclonal EpoR antibody (clone 3D10).

The localization of EpoR by immunohistochemistry confirmed previous studies on chemically induced hepatocellular carcinoma in mice (Nakamatsu et al. 2004), indicating the presence of the receptor in the sinusoids within the regenerative nodules and supporting an important role of EPO in the vasculogenesis and angiogenesis related to disturbances of the circulatory tree in the cirrhotic liver that are crucial for the expansion and proliferation of the tumor. On the other hand, CC cells localized in the hyperplastic biliary ducts also showed detectable amounts of EpoR as demonstrated in the co-localization staining with CK19. Furthermore, we found high levels of EpoR transcripts in isolated Kupffer cells, and isolated clusters of ED-2 positive cells in TAA-treated livers showed signals of positivity for the EpoR antibody. Although it seems immature to draw conclusions in this direction, a recent observation by Broxmeyer (2011) might suggest a possible role for EPO in the re-organization of the tumor-associated immune response (Broxmeyer 2011).

To elucidate the functional role of EPO/EpoR signaling in cell proliferation and/or cell survival, we analyzed the effects of EPO stimulation on genes involved in the cell cycle, such as PCNA and CyclinD1, as previously observed in other cancer cell lines (Feldman et al. 2006). Upon binding to its receptor, EPO leads to a dimerization and transphosphorylation which triggers proliferating and anti-apoptotic pathways. This in turn initiates additional specific signal transduction pathways, including the phosphatidylinositol 3-kinase (PI-3K)/AKT pathway, MAPKs family member cascade and the JAK/STAT pathway, which are fundamental not only for hepato-protective signaling, but also in liver regeneration and repair (Gao 2005) Indeed, in Mz-Cha-2, the cell line with the highest EPO gene expression, a modest but dose-dependent increase of the two downstream genes was observed under normoxic conditions.

Finally, referring to previous reports from our group (Mansuroglu et al. 2009) and others (Kamenz et al. 2006) showing an aberrant expression of the SCF/-c-kit pathway in the same experimental model, a synergistic effect of SCF and EPO on cell functions was hypothesized. In particular, the cell populations that express the highest levels of c-kit receptor, hepatocytes of the cirrhotic nodules and hyper-proliferative CK19-positive cells, were found to be the same cell types that contribute to the greatest extent to EPO production. Moreover, during erythropoiesis and in the fetal liver, the crosstalk between these growth factors has been shown to induce proliferation and differentiation of erythroid progenitor cells through signals commonly triggering JAK2/STAT5 phosphorylation and activation (Wu et al. 1997; Arcasoy and Jiang 2005). The synergistic effect of EPO and SCF on EPO production observed under normoxic condition in Mz-Cha-2 cells, with a concomitant increase of EPO gene expression, PCNA and CyclinD1, indicates a sensitization to EPO responsiveness as already observed in another cancer cell line (Sato et al. 1998). This result might offer new evidence for a possible, hypothesized hematopoietic origin of CC cancer cells (Cardinale et al. 2012).

In conclusion, evidence of an efficacious proliferative challenge for tumor cells remains elusive and requires further in vitro and in vivo investigations (Aapro et al. 2012). We described new tumor cell populations that are able to synthesize EPO in vivo and in vitro, but respond differently to stimulation. This study reaffirmed the importance of EPO as a molecular mediator produced by the liver during the process of chronic hepatic injury and regeneration in its progression to cancer.

## Electronic supplementary material

Below is the link to the electronic supplementary material.

**Supplementary Fig. 1.** Different EpoR isoforms and specific binding sites recognized by the antibodies used in the present study. The full-length peptide is composed by an intracellular domain (gray) that is recognized by the Santa Cruz Antibody M-20. Through alternative splicing other two receptor isoforms can originate: a soluble isoform, EpoR-s, that can be systemically released and a truncated form, EpoR-t, lacking a part of the intracellular domain. The 3D10 antibody, binding the extracellular domain (in pink and red), enables the detection of all the three receptor isoforms. (TIFF 154 kb)
