# Supplementary figures and images for: Characterization of the erythropoietin/erythropoietin receptor axis in a rat model of liver damage and cholangiocarcinoma development
Source: Histochem Cell Biol. 2012 Oct 4;139(3):473–85. doi: 10.1007/s00418-012-1037-x (PMC3573187; doi:10.1007/s00418-012-1037-x)

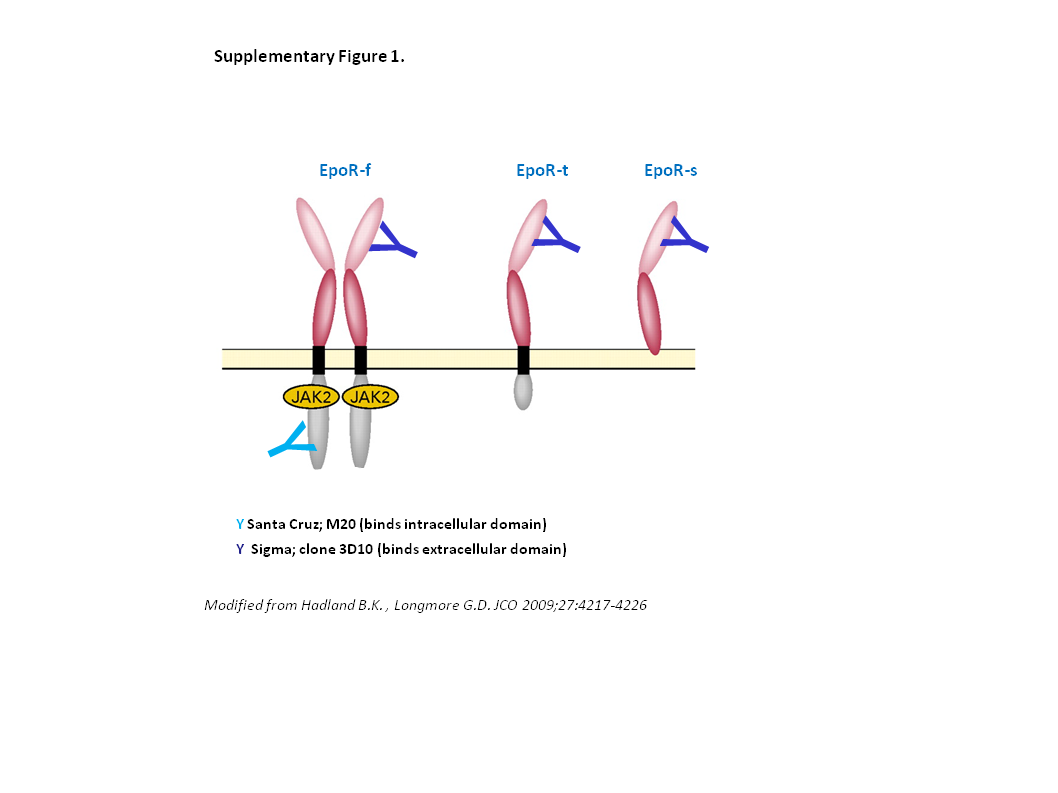

Supplement: Supplementary file 1 — Supplementary Fig. 1. Different EpoR isoforms and specific binding sites recognized by the antibodies used in the present study. The full-length peptide is composed by an intracellular domain (gray) that is recognized by the Santa Cruz Antibody M-20. Through alternative splicing other two receptor isoforms can originate: a soluble isoform, EpoR-s, that can be systemically released and a truncated form, EpoR-t, lacking a part of the intracellular domain. The 3D10 antibody, binding the extracellular domain (in pink and red), enables the detection of all the three receptor isoforms. (TIFF 154 kb) [file 418_2012_1037_MOESM1_ESM.tif]
